# Minimally invasive plate fixation of paediatric lower limb long bone fractures: a review on when and how

**DOI:** 10.1530/EOR-2025-0122

**Published:** 2026-04-07

**Authors:** Alberto Daniel Navarro Vergara, Jamil Soni, Weverley Rubele Valenza, Estefanía Birrer, Heloisa Zimmmermann Faggion

**Affiliations:** ^1^ Hospital de Trauma “Manuel Giagni”, Avenida Gral Máximo Santos, Asunción, Paraguay; ^2^ Universidad del Norte, Avenida España No. 676, Entre Rosa Peña y Boquerón, Asunción, Paraguay; ^3^ Universidad Nacional de Itapua, María Auxiliadora, Paraguay; ^4^ Hospital do Trabalhador, Avenida República Argentina, Curitiba, PR, Brazil; ^5^ Hospital Universitário Cajuru, Avenida São José, Curitiba, PR, Brazil; ^6^ Pontifícia Universidade Católica do Paraná, Curitiba, PR, Brazil; ^7^ Hospital Base de Valdivia, Valdivia, Los Ríos, Chile; ^8^ Universidad Austral de Chile, Independencia, Valdivia,Los Ríos, Chile; ^9^ Universidade Federal de Paraná, Curitiba, PR, Brazil

**Keywords:** femur, paediatrics, trauma, surgery

## Abstract

- Fractures of the lower limbs represent a common cause of hospital admission.
- Surgical intervention is increasingly indicated in paediatric patients.
- The gold standard for treating long bone fractures in the immature skeleton is intramedullary fixation with titanium elastic nails.
- Minimally invasive plating is considered a suitable option when elastic nailing is not feasible.
- The use of a plate provides adequate mechanical stability for weight-bearing and limb mobility while preserving biological bone integrity and resulting in limited scarring, generally well accepted by patients and families, although it requires a more extensive incision than intramedullary fixation.
- Potential drawbacks of this technique include a more extensive surgical approach, possible delay in weight-bearing, and the potential need for implant removal.

## Introduction

Musculoskeletal injuries of the lower limbs are among the leading causes of emergency hospital admissions in children (1). Approximately 25% of these injuries involve fractures, most commonly of the tibia (2). Nearly 50% of children will sustain a fracture before adulthood, occasionally resulting in long-term consequences (1, 2). Paediatric fractures differ substantially from those in adults, requiring tailored management due to variations in bone structure, healing time, and emotional context (3).

Surgical intervention is increasingly indicated in paediatric trauma, particularly for femoral fractures in children over 4 years of age, whereas conservative management remains the standard of care for most tibial fractures, with surgery reserved for selected indications. Elastic stable intramedullary nailing (ESIN) has become the gold standard for treating paediatric lower limb long bone fractures, but its widespread use has been associated with specific complications (3). In this context, bridge plating with minimally invasive plate osteosynthesis (MIPO) has emerged as a viable alternative for fractures with a high risk of non-union following ESIN, particularly when instability, patient weight, or fracture site preclude intramedullary fixation (3, 4). While MIPO can be applied to different long bones, its use in children is most common for femoral shaft fractures, with tibial applications being less frequent and reserved for selected cases. Despite addressing some limitations of ESIN, MIPO presents its own challenges, such as difficulties related to implant removal and a steep learning curve (4).

This study aims to outline the indications for bridge plating and describe the surgical technique employed in paediatric patients with diaphyseal fractures of the femur and tibia. As most published studies report functional outcomes qualitatively, typically as ‘good’ or ‘excellent’, without standardised scoring systems, this review focuses on the technical aspects and reported outcomes of the MIPO approach. This study also seeks to evaluate the effectiveness of this approach to further support paediatric orthopaedic surgeons in decision-making, thereby contributing to improved quality of care for children.

## Rationale for the use of bridge plating

Surgical treatment of long bone fractures of the lower limb in children remains a subject of debate. Multiple factors influence the choice of treatment, including patient age and weight, fracture pattern, the specific diaphyseal region involved, soft tissue condition, associated injuries, surgeon experience, and implant availability (5). Established surgical options include external fixation, intramedullary nailing, and plate-and-screw fixation. Regardless of the chosen method, the purpose is to employ a minimally invasive approach that preserves bone length, avoids rotational or angular deformities, and preserves bone integrity through biological internal fixation (6).

Biological internal fixation aims to maintain relative stability at the fracture site rather than absolute rigidity, thereby stimulating callus formation and promoting bone healing. This principle underpins the rationale for bridge plating, which provides stable fixation while preserving the vascularity of bone fragments and minimising periosteal disruption (5, 6). This technique offers advantages comparable to those of rigid nailing, while avoiding complications such as physeal injury, avascular necrosis, and deformities including coxa vara. Furthermore, it mitigates risks associated with elastic nailing and external fixation.

Traditionally, MIPO plating has been indicated for diaphyseal fractures of long bones. However, it can also be beneficial in selected metaphyseal fractures, provided that the growth plate is not compromised. Bridge plating facilitates early mobilisation, promotes functional independence, prevents malalignment and limb shortening, and supports biological bone healing and optimal long-term remodelling. It is considered a reproducible technique with a short learning curve (7).

## Indications

While patient age and weight are key factors in selecting the treatment for long bone fractures of the lower limb, several additional factors must also be considered when choosing the appropriate implant, including bone exposure, polytrauma, associated injuries, and proximal or distal metaphyseal involvement. Defining a fracture as stable or unstable based on its pattern can help determine the appropriate treatment pathway. According to Kocher *et al.* (8), comminuted, spiral, and long oblique fractures should be classified as unstable, requiring particular attention when planning fixation.

The interplay of these factors renders the selection of an appropriate surgical strategy both challenging and complex. Although MIPO can be a valuable option for selected unstable or comminuted tibial fractures, most paediatric tibial fractures continue to be treated conservatively, and surgical fixation is reserved for specific situations, such as open fractures, polytrauma, or failed closed management.

## Results of the use of bridge plating

The literature consistently supports the use of submuscular bridge plating for the management of unstable long bone fractures in the paediatric population (3, 4, 7). This technique has demonstrated effectiveness across all forms of diaphyseal fractures, including those resulting from high-energy trauma, spiral fracture patterns, or fractures located adjacent to the bone ends, as well as in cases in which titanium elastic nails are unsuitable due to patient weight or size (7).

Additional indications include scenarios in which rigid nails for adolescents are unavailable. In femoral fractures, the lateral entry approach is preferred to avoid compromising the piriform fossa or the proximal femoral physis. For tibial fractures, rigid nails are inserted proximal to the anterior tibial tuberosity; consequently, their use is contraindicated in patients with open growth plates.

Bridge plating provides adequate mechanical stability for weight-bearing and limb mobility while preserving biological bone integrity and resulting in limited scarring, generally well accepted by patients and families. This technique also facilitates restoration of proper alignment and limb length with minimal trauma to the fracture site.

In a multicentre study involving 51 paediatric femoral fractures treated with submuscular plating, Kanlic *et al.* (7) reported uniformly good to excellent outcomes, with few technique-related complications. The technique was associated with early mobilisation without the need for postoperative immobilisation, prevention of malalignment and limb shortening, excellent bone healing, and uncomplicated implant removal. The authors highlighted the technique’s safety and reproducibility, while recommending against the use of 3.5 mm plate systems or titanium plates for definitive fixation (7, 8). Similarly, Ağuş *et al.* (5) concluded that bridge plating represents a biologically sound alternative for the management of femoral fractures, especially those near the bone ends. In their series of 14 cases, both clinical and radiographic outcomes were consistently satisfactory (5).

Regarding tibial applications, Masquijo (9) reported on 11 patients with distal metaphyseal fractures treated with percutaneous plating. The mean time to union was 13 weeks, and no major complications occurred. Minor superficial skin irritation was observed due to plate contact, but no rotational deformity, leg-length discrepancy, or physeal growth arrest was identified. The author recommended the use of plating in older children and adolescents, citing the advantages of early mobilisation and stable fixation through a minimally invasive approach (9). Özkul *et al.* (10) investigated the effectiveness of plating in open tibial fractures and did not recommend its use as the first-line therapy. Instead, they suggested restricting its use to selected adolescent cases. In their series of 14 patients, lateral plating, whether using anatomical or straight plates, was not associated with major complications or detrimental impact attributable to the implant design (10).

## Complications with bridge plating

Conventional compression plate fixation has historically been used in paediatric patients, particularly in those with multiple injuries (11, 12, 13). However, disruption of fracture biology caused by this technique has been associated with early implant failure, and therefore, its use is not recommended. In addition, cases of excessive bone growth have been reported following conventional compression plating (12).

Bridge plating in paediatric patients has raised concerns due to its association with progressive deformity. The risk of valgus deformity increases when plates are positioned within 2 cm of the physis, as this proximity is more likely to disturb bone alignment (14). Implant removal at approximately 12 months postoperatively appears to mitigate this risk (15), owing to residual bone growth potential and the disruptive effect of the plate on femoral remodelling.

Submuscular bridge plating offers advantages over conventional compression plate fixation, including more rapid and reliable bone healing, as it preserves the biological environment and avoids direct exposure of the fracture site. Minimal soft tissue dissection also contributes to reduced postoperative pain, enhanced scarring, faster functional recovery, and improved cosmesis (7, 16). In a multicentre series, Kanlic *et al.* (7) reported two major complications (one plate failure and one refracture), both attributed to preventable technical errors: inappropriate plate selection and premature implant removal in an undiagnosed pathological fracture (7).

**Figure 1:**
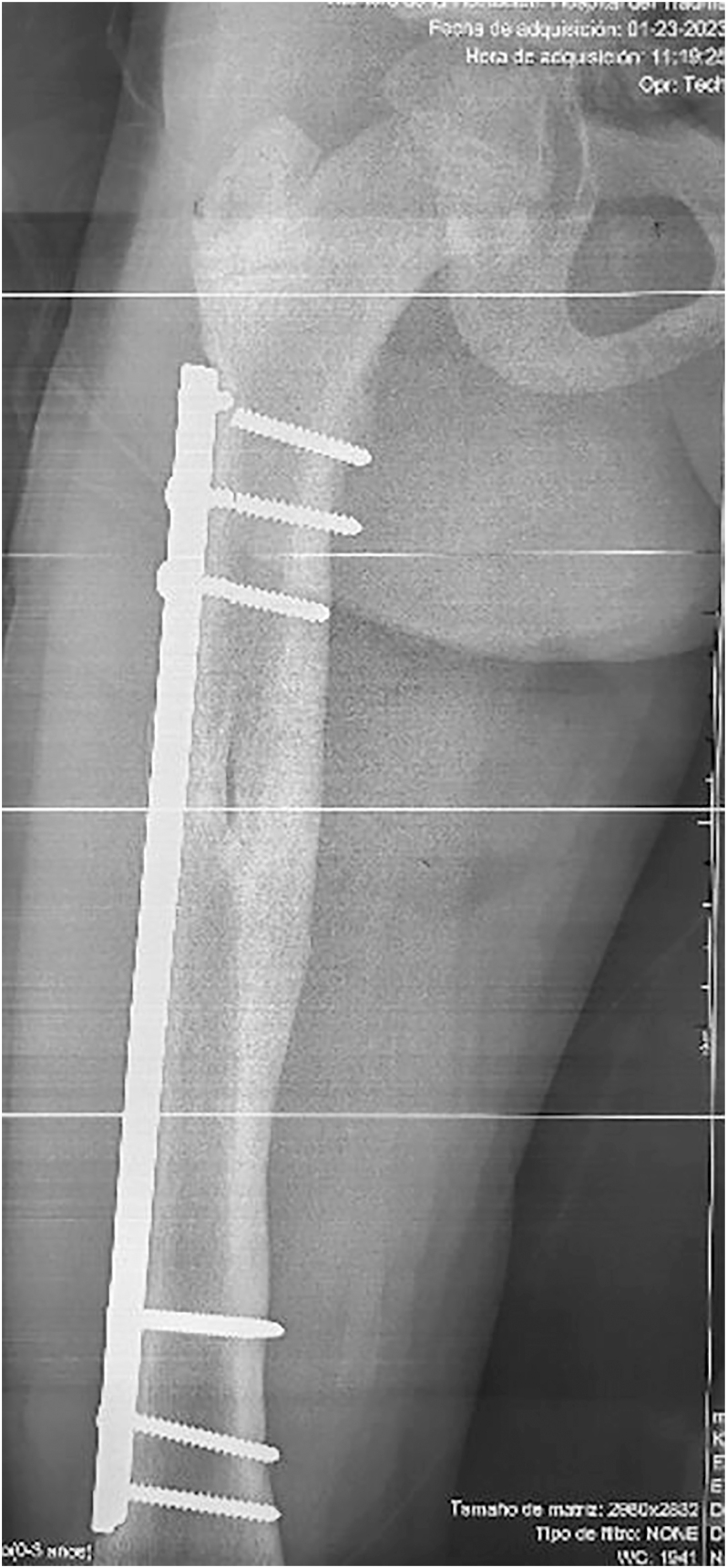
Anteroposterior radiograph showing implant failure after 12 months *in situ* due to breakage of the three proximal screws.

Plate retention in patients with substantial residual growth potential is not recommended. Reported plate-related complications include proximal migration of the implant, valgus deformity, and medial protrusion of screws at the fixation site (Fig. 1) (17, 18). In tibial fractures, common complications include infection in open fractures, compartment syndrome due to high-energy trauma, and residual leg-length discrepancy after healing (9, 10). These complications are uncommon with bridge plating, since the plate is positioned away from the exposed bone. The technique is generally employed in older children and adolescents, a relevant factor in mitigating the risk of compartment syndrome, and careful avoidance of the physis during screw insertion reduces the likelihood of premature physeal closure or angular deformity.

**Figure 9:**
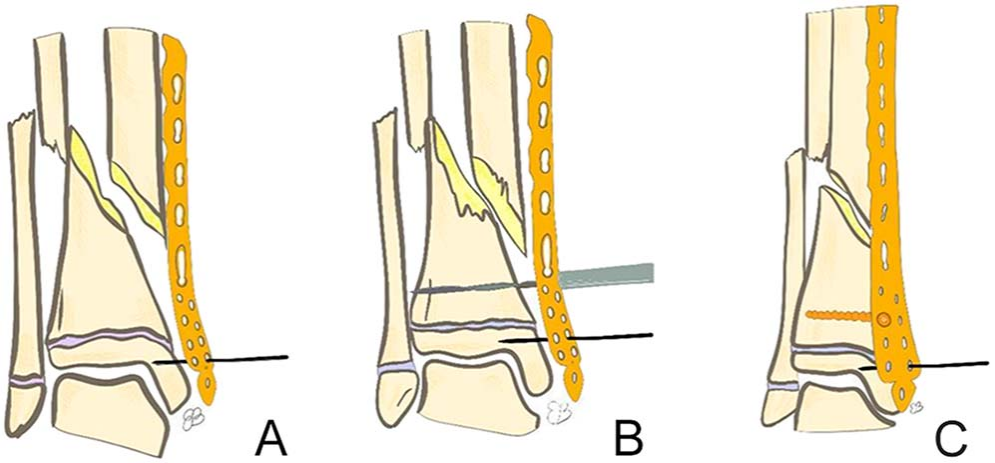
Images illustrating screw placement and fracture reduction via the plate (A, B, C).

One specific concern is skin irritation in the distal third of the leg, where subcutaneous plate prominence may lead to local discomfort or, in severe cases, hardware exposure (9, 10). Other potential drawbacks of the MIPO technique include a more extensive surgical approach, possible delay in weight-bearing, and the potential need for implant removal.

Contraindications to the use of MIPO include intra-articular fractures, injuries extending into the epiphysis, upper-limb fractures in paediatric patients, and cases involving severe soft tissue damage unless this has been adequately managed before fixation (18, 19).

## Authors’ preferred technique

### Femoral fractures

Preoperative planning begins with radiographic evaluation, using the contralateral femur as a reference to determine plate tension and length, based on proximal and distal screw positioning. The preferred implant is a 4.5 mm narrow locking compression plate (LCP) or limited-contact dynamic compression plate, which provides adequate bone support, allows precise contouring at the proximal and distal ends while maintaining straight alignment between them, and enables screw placement through minimally invasive or percutaneous approaches.

**Figure 2:**
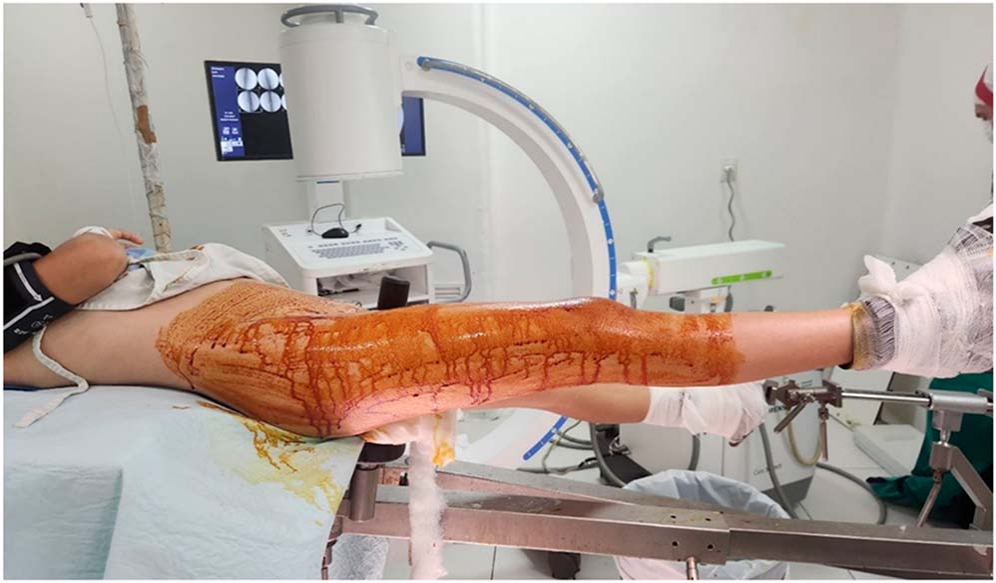
Patient positioned on a traction table enabling correction of misalignment through continuous traction.

**Figure 3:**
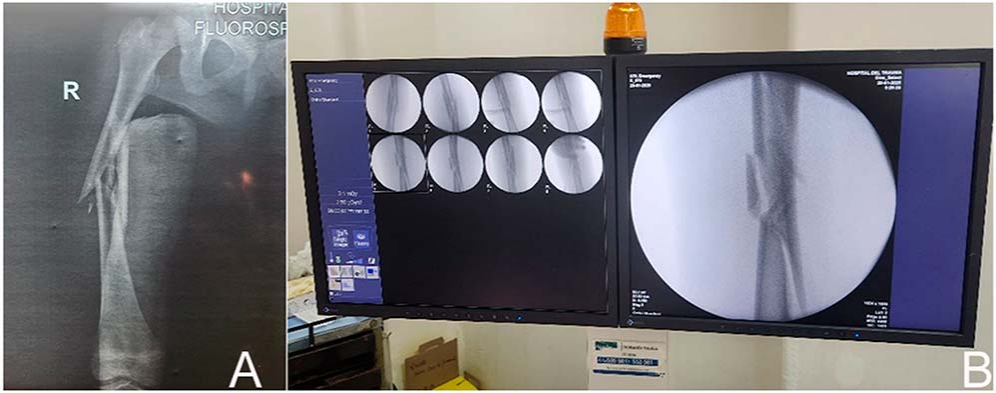
Preoperative radiograph (A) and fluoroscopic image following limb fracture (B).

**Figure 4:**
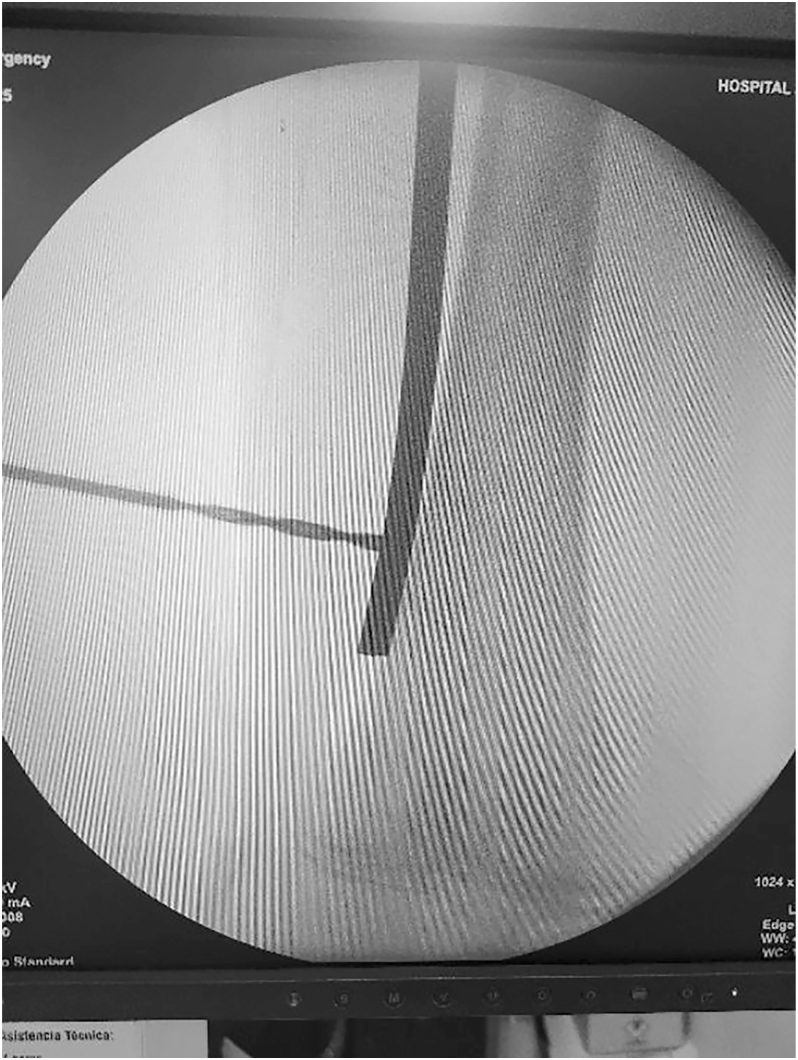
Insertion of a 3.2 mm drill bit for provisional fixation of the plate to the bone.

The patient is positioned on either a traction or radiolucent table (Fig. 2), depending on the fracture location and surgeon preference. This setup facilitates restoration of limb length and correction of misalignment (Fig. 3). Incision sites are marked preoperatively. The distal incision is made first, exposing the thin distal portion of the vastus lateralis. Blunt dissection is performed with a retractor to separate the muscle from the periosteum, followed by careful curettage. The plate is inserted beneath the vastus lateralis and above the periosteum, ensuring continuous contact between the proximal end of the plate and the femoral shaft as it is gradually advanced proximally. Fluoroscopic guidance assists with accurate positioning. Once in place, a Kirschner wire (or, alternatively, a 3.2 mm drill bit) may be inserted through the first distal hole for temporary plate fixation. Implant length is confirmed fluoroscopically before the proximal surgical approach (Fig. 4).

**Figure 5:**
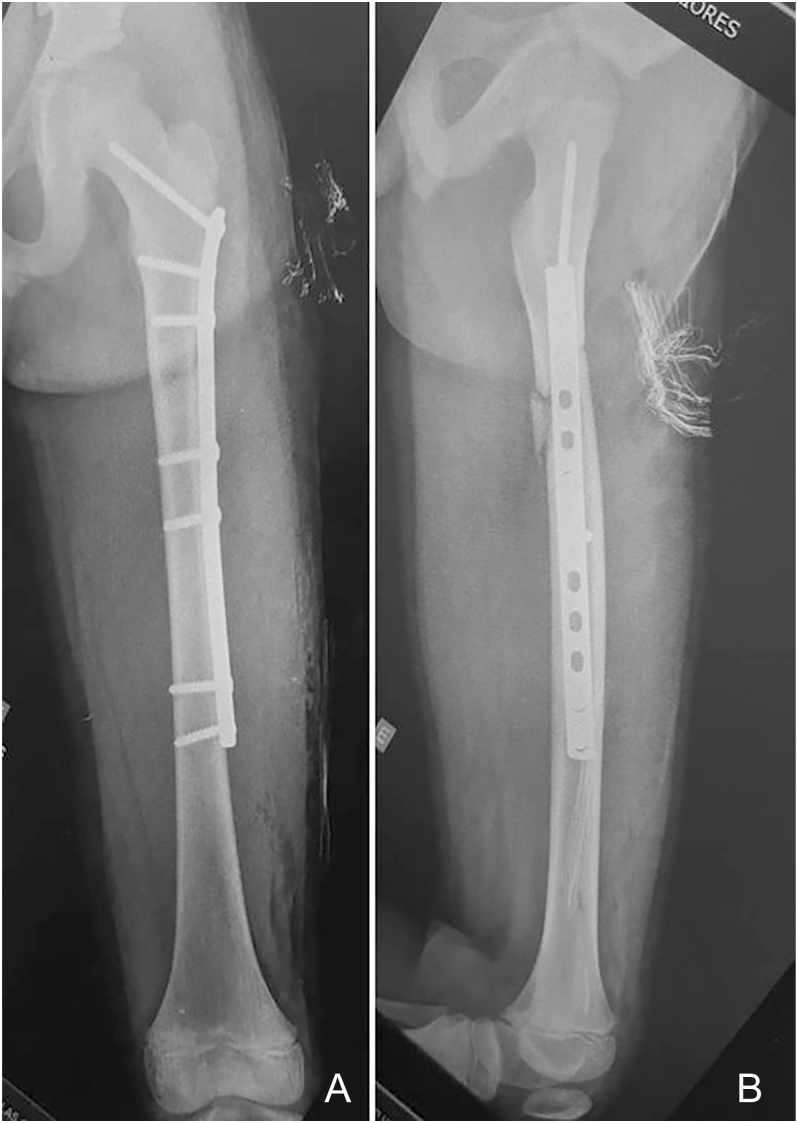
Radiographs showing screws arranged in the dispersed pattern, also known as near–near, far–far pattern: anteroposterior view (A); lateral view (B).

**Figure 6:**
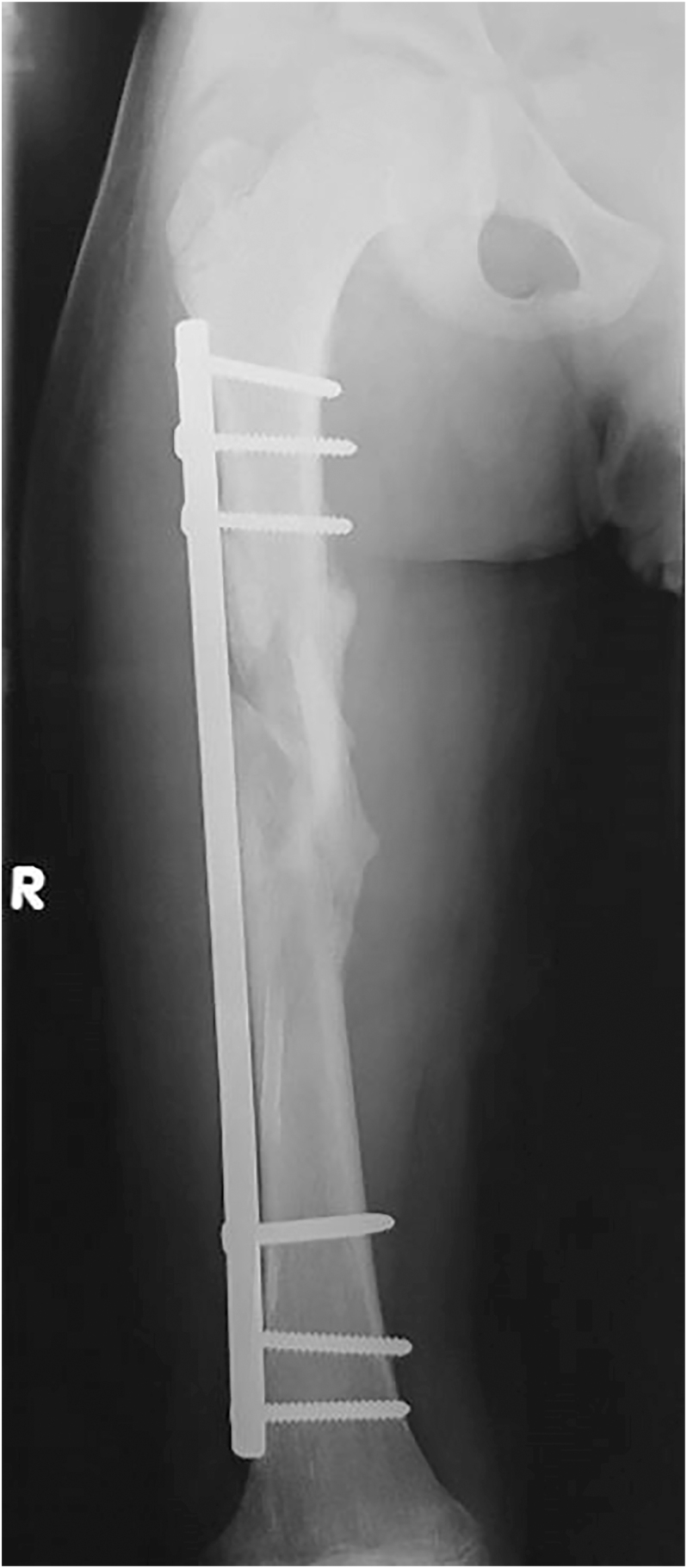
Radiograph showing a clustered screw pattern, with screws placed at both ends of the plate.

Based on biomechanical studies, Rozbruch *et al.* (16) recommended an even distribution of screws, analogous to external fixation, with screws positioned at the end of the main fragment, close to the fracture, and at mid-fragment. This configuration, labelled as ‘dispersed screw pattern’ (Fig. 5), provides relative stability by maximising the distance between screws in each fragment – a configuration referred to as ‘near–near, far–far pattern’ (i.e. one at the end of the plate and one near the fracture line) (7, 16). Alternatively, the so-called ‘clustered screw pattern’ involves bicortical fixation at both ends of the plate, reducing the number of incisions required while maintaining stability comparable to that of intramedullary nailing (Fig. 6) (7).

In our practice, screws are placed close to the fracture site on both the proximal and distal sides. Fixation typically begins with the fragment most distant from the plate to help approximate the bone to the implant. Distal screws are placed under direct vision through the initial incision. Accurate drilling is ensured by fluoroscopic visualisation of the plate holes as perfect circles (20). Small incisions are made through the skin, iliotibial band, and vastus lateralis at the intended screw sites, followed by freehand drilling with a 3.2 mm drill bit under fluoroscopic guidance (7, 20). To prevent loss of screw control during percutaneous insertion, a Vicryl suture may be tied around the screw head (7). The use of self-drilling screws is recommended to avoid threading into soft tissues. Fixation is completed by inserting screws both proximally and distally until a minimum of six cortices are secured (three screws on each side of the fracture). Optimal screw distribution enhances construct stability and obviates the need for additional screws (20).

### Tibial fractures

**Figure 7:**
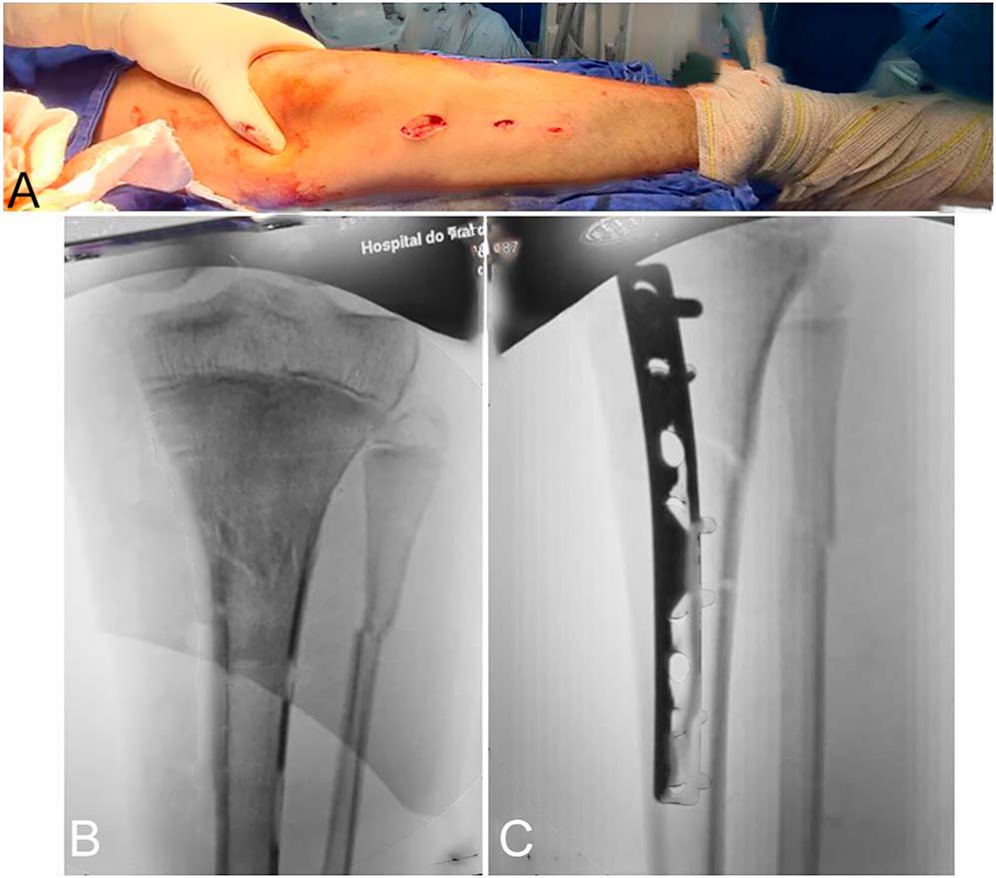
Images showing skin incisions (A) and a pre-contoured straight plate designed to improve contact with the bone (B and C).

Radiographic assessment of the contralateral limb guides implant selection. Depending on fracture location, either pre-contoured plates for proximal or distal tibial fractures or straight plates similar to those used in femoral fixation may be selected (Fig. 7). Plate positioning on the tibia is dictated by fracture configuration and soft tissue condition: medial plating is preferred for distal and mid-shaft fractures, whereas lateral plating is favoured for proximal fractures.

**Figure 8:**
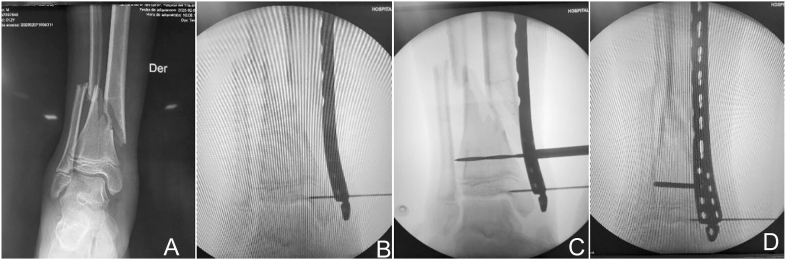
Preoperative anteroposterior radiograph (A), insertion of a temporary pin to hold the plate in place (B), bone drilling with a drill bit (C), and fracture reduction using a screw to approximate the bone fragment to the plate (D).

The patient is positioned supine on a radiolucent table; a tourniquet is not used (10). Skin markings are made according to the preoperative plan. Fracture reduction is achieved through traction and counter-traction techniques. Once satisfactory alignment is confirmed, distal incisions are made, followed by gentle muscle dissection to avoid periosteal injury. The plate is inserted in a retrograde manner and positioned relative to the bone while maintaining reduction. Temporary fixation is achieved with Kirschner wires, and the screw closest to the fracture is inserted at the most distal point on the plate to ensure proper implant positioning (Figs 8 and 9) (9).

The procedure is completed by placing the remaining proximal and distal screws. Final radiographs are obtained to confirm alignment and fixation, consistently assessing both views. Surgical wounds are then closed. Plate selection corresponds to fracture location, with 4.5 mm LCPs typically used for fixation (9, 10).

### Postoperative care

Postoperative cast or splint immobilisation is unnecessary. All patients are followed for a minimum of 2 years post-injury.

For femoral fractures, once fixation and reduction are confirmed radiographically, early mobilisation of the hip and knee is initiated during hospitalisation. Partial weight-bearing with crutches is recommended for approximately 6 weeks, progressing to full weight-bearing upon radiographic evidence of bone union. Plate removal is recommended approximately 9 months postoperatively, once healing of at least three cortices and canal recanalisation are evident, to prevent excessive bone overgrowth, which may hinder plate removal. During removal, a Cobb elevator can be inserted from the distal end to elevate the vastus lateralis and gently separate the plate from the periosteum (20).

For tibial fractures, early joint mobilisation is encouraged, with non-weight-bearing maintained for 4–6 weeks, depending on recovery. Gradual progression to full weight-bearing is introduced to stimulate bone healing and remodelling (6).

## Conclusion

Fractures of the lower limb are common in the paediatric population and, when appropriately indicated, may be treated surgically using different operative methods. Bridge plate fixation represents a versatile technique that paediatric orthopaedic surgeons should be familiar with for the effective management of these fractures.

Bridge plating is indicated for diaphyseal comminuted fractures, metaphyseal fractures, and unstable fracture patterns. It is also suitable for patients with increased body weight and narrow medullary canals or when appropriate implants, such as adolescent-specific rigid nails, are unavailable. The technique involves closed or minimally invasive reduction, fixation with a contoured or anatomical plate, and screw placement in a dispersed or clustered pattern. Once fracture union is achieved, implant removal is recommended.

## ICMJE Statement of Interest

The authors declare that there is no conflict of interest that could be perceived as prejudicing the impartiality of the work reported.

## Funding Statement

This work did not receive any specific grant from any funding agency in the public, commercial, or not-for-profit sector.

## Author contribution statement

ADNV conceived the study, designed the methodology, performed formal analysis, administered the project, acquired resources and software, and wrote the original draft of the manuscript; ADNV, JS, and WRV curated the data, performed validation, and supervised the study; ADNV, JS, WRV, and HZF were involved in the investigation; ADNV, JS, and WRV supervised the study; EB performed visualisation; ADNV, JS, WRV, and EB reviewed and edited the manuscript.
